# Comparison of trans-umbilical single-port laparoscopic complete extraperitoneal closure and laparoscopic intracorporeal closure for pediatric inguinal hernia: a randomized controlled study

**DOI:** 10.3389/fped.2024.1509895

**Published:** 2024-12-23

**Authors:** Yanyi Li, Zhu Jin, Chengyan Tang, Yuan Gong, Lu Huang, Qing Du, Xinrong Xia, Daiwei Zhu, Wankang Zhou, Zeping Li, Weiao Wang, Yuanmei Liu, Zebing Zheng

**Affiliations:** Department of Pediatric Surgery, Children Hospital of Guizhou Province, Affiliated Hospital of Zunyi Medical University, Zunyi, Guizhou, China

**Keywords:** complete extraperitoneal closure, inguinal hernia, intracorporeal closure, laparoscopy, pediatric surgery

## Abstract

**Background:**

The purpose of this study was to compare the outcomes of Trans-umbilical single-port laparoscopic complete extraperitoneal closure (LCEC) and laparoscopic intracorporeal closure (LIC) for inguinal hernia by analysis of follow-up data over 5 years.

**Methods:**

In this prospective randomized controlled trial, 524 children with inguinal hernia were randomly assigned to undergo LCEC or LIC between August 2016 and December 2017. The primary outcome measures were the success and recurrence rates. The secondary outcome measures were operative time; length of hospital stay; postoperative pain score; and incidence of postoperative complications, including rates of wound infection, stitch abscess, and testicular atrophy.

**Results:**

Primary analysis of the 227 patients in the LIC group and 215 patients in the LCEC group revealed that in the LCEC group, the success rate of was significantly higher in LCEC group (96.7% vs. 90.3%, *P* < .05) and the length of hospital stay was significantly shorter (*P* < .05) than those of the LIC group. Neither the recurrence rate (*P* > .05) nor the operative time (*P* > .05) of the groups significantly differed. The pain scores at postoperative 12 and 24 h were significantly lower in the LCEC group than in the LIC group (*P* < .05). The incidence rates of wound infection (0.93% vs. 5.7%, *P* < .05) and stitch abscess (1.4% vs. 7.0%, *P* < .05) were significantly lower in the LCEC group than in the LIC group. No testicular atrophy occurred in either group.

**Conclusion:**

LCEC is associated with better clinical success and fewer postoperative complications for repair of pediatric inguinal hernia compared with LIC.

## 1 Introduction

In pediatric surgery, inguinal hernia is one of the most common surgical diseases, with incidence rates ranging from 1% to 4% (1). Inguinal herniorrhaphy has traditionally been treated by open surgery for more than 100 years, with recent studies reporting recurrence rates ranging from 0% to 6% (2, 3). With the development of laparoscopic procedures, laparoscopic inguinal hernia closure has gained increased popularity, and the use of inguinal hernia repair techniques has been reported (4). Laparoscopic procedures for patent processus inguinal hernia closure, which use both intracorporeal and extracorporeal methods, have become widely accepted as the most simple and effective procedures for pediatric inguinal hernia closure (5, 6).

As the use of the trocar decreases, the use of extracorporeal knotting techniques continues to increase and evolve. We introduced trans-umbilical single-port laparoscopic complete extraperitoneal closure (LCEC) for pediatric inguinal hernia at our institution in October 2014 as a less invasive and more cosmetically appealing procedure (7). Requiring only one port with laparoscopic vision, LCEC uses a two-hook hernia needle with a non-absorbable suture inserted at the abdominal transverse striation to the extraperitoneal space without puncture of the peritoneum, followed by ligation and closure of the orifice of the inner hernia. By such means, LCEC preserves the integrity of the peritoneum, an advantage compared with trans-umbilical single-port laparoscopic intracorporeal closure (LIC).

Few studies have compared conventional open surgery and laparoscopic surgery for inguinal hernia or compared intracorporeal closure and extracorporeal closure laparoscopic procedures (8). There is insufficient evidence to conclude that laparoscopic extracorporeal closure provides long-term advantages, particularly when comparing intracorporeal and completely extracorporeal procedures. To fill this research gap, we tested the hypothesis that LCEC is superior to LIC for the treatment of pediatric inguinal hernia by conducting a prospective non-inferiority randomized controlled trial (RCT).

## 2 Methods

This trial was registered at ClinicalTrials.gov (ClinicalTrials.gov identifier: NCT02960529) before initiation and designed as a prospective RCT. This study was approved by the regional Ethics Committee for Medical Research of Zunyi Medical University (Approval No. 2016090138) and conducted following the Declaration of Helsinki. Written informed consent to participate in the study was obtained from all patients' parents/guardians.

All consecutive patients with inguinal hernia who were surgically treated at the Affiliated Hospital of Zunyi Medical University from August 2016 to December 2017 were screened for inclusion by research staff. Surgical procedures are performed by attending physicians with over 3 years of experience or higher-level professional title physicians. The inclusion criteria were treatment for unilateral hernia, bilateral hernia, or hernia with hydrocele; age 6 months to 13 years; male sex; and clinical diagnosis of inguinal hernia according to clinical presentation and ultrasound. Exclusion criteria were ascending testis, recurrent hernia, incarcerated hernia, inguinal hernia with a history of abdominal surgery, and inguinal hernia combined with severe cardiopulmonary diseases.

Stata software (https://www.stata.com) was used to generate random grouping numbers for randomization in a 1:1 allocation ratio the day before surgery. Each randomized patient was given a unique study ID number. Based on the literature, the incidence of recurrence in the LIC group was assumed to be 3% and the incidence in the LCEC group 0.5% (7, 9). Using a two-sided *P* value of.05 and test power (1 − *β*) of 80%, the optimal sample size was calculated to be 428. Considering that 10% of the patients might be lost to follow-up, at least 470 patients were needed for this study.

### 2.1 LIC procedure

**Figure 1:**
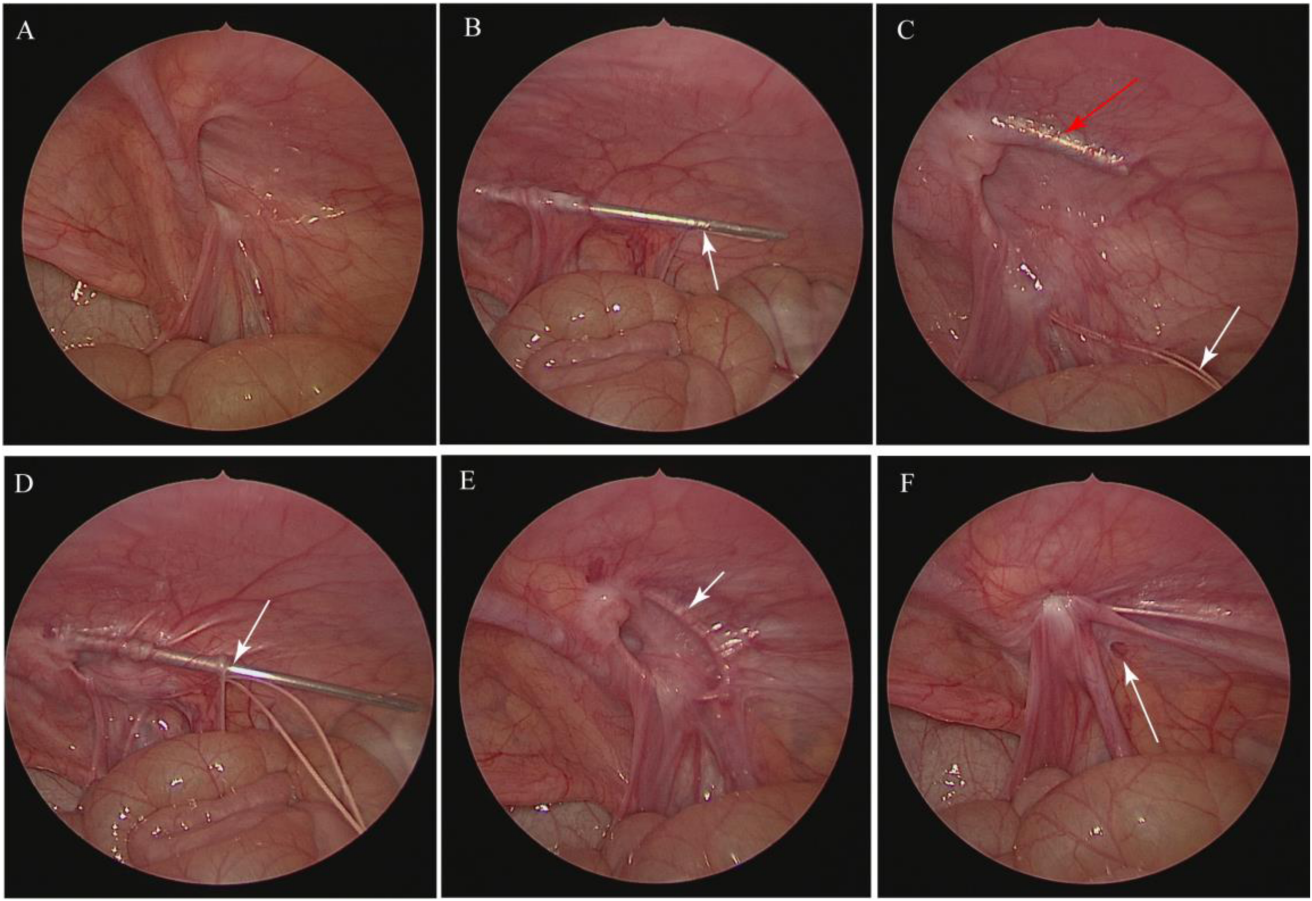
Steps in the LIC procedure for inguinal hernia. **(A)** The general condition of the inner ring was explored by laparoscopy. **(B)** The peritoneum was punctured by the hernia needle, as indicated by the arrow. **(C)** After the suture was placed in the peritoneum, as indicated by the white arrow, the hernia needle was inserted along the lateral half side of the internal ring, as indicated by the red arrow. **(D)** The hernia needle was passed through the peritoneal puncture point to the abdominal cavity, as indicated by the arrow, before the suture was pulled out of the abdominal cavity. **(E)** Ligation and closure of the orifice of the inner hernia were performed, as indicated by the arrow. **(F)** Inner ring closure after ligation was performed. The rupture on the peritoneum is indicated by the arrow.

The LIC used to repair inguinal hernia was that described previously by Zhang et al. (7) but revised to include one trocar in the umbilicus. A 5 mm incision was made around the umbilicus for the laparoscopic light source. A 1 mm incision was made at the surface projection point of the inner ring, and a 3/0 non-absorbable suture was inserted through the preperitoneal space by the hernia needle through the incision (Figure 1A). The hernia needle was used to puncture the peritoneum (Figure 1B) and place the suture in the peritoneum until it passed through the vas deferens and spermatic cord blood vessels (Figure 1C). After the hernia needle was returned to the outer peritoneal space at the top of the hernia sac, it was inserted along the lateral half side of the internal ring through the peritoneal puncture point to the abdominal cavity before the suture was pulled out of the abdominal cavity (Figure 1D). Finally, ligation and closure of the orifice of the inner hernia were performed under assisted laparoscopy without the use of grasping forceps (Figures 1E,F).

### 2.2 LCEC procedure

**Figure 2:**
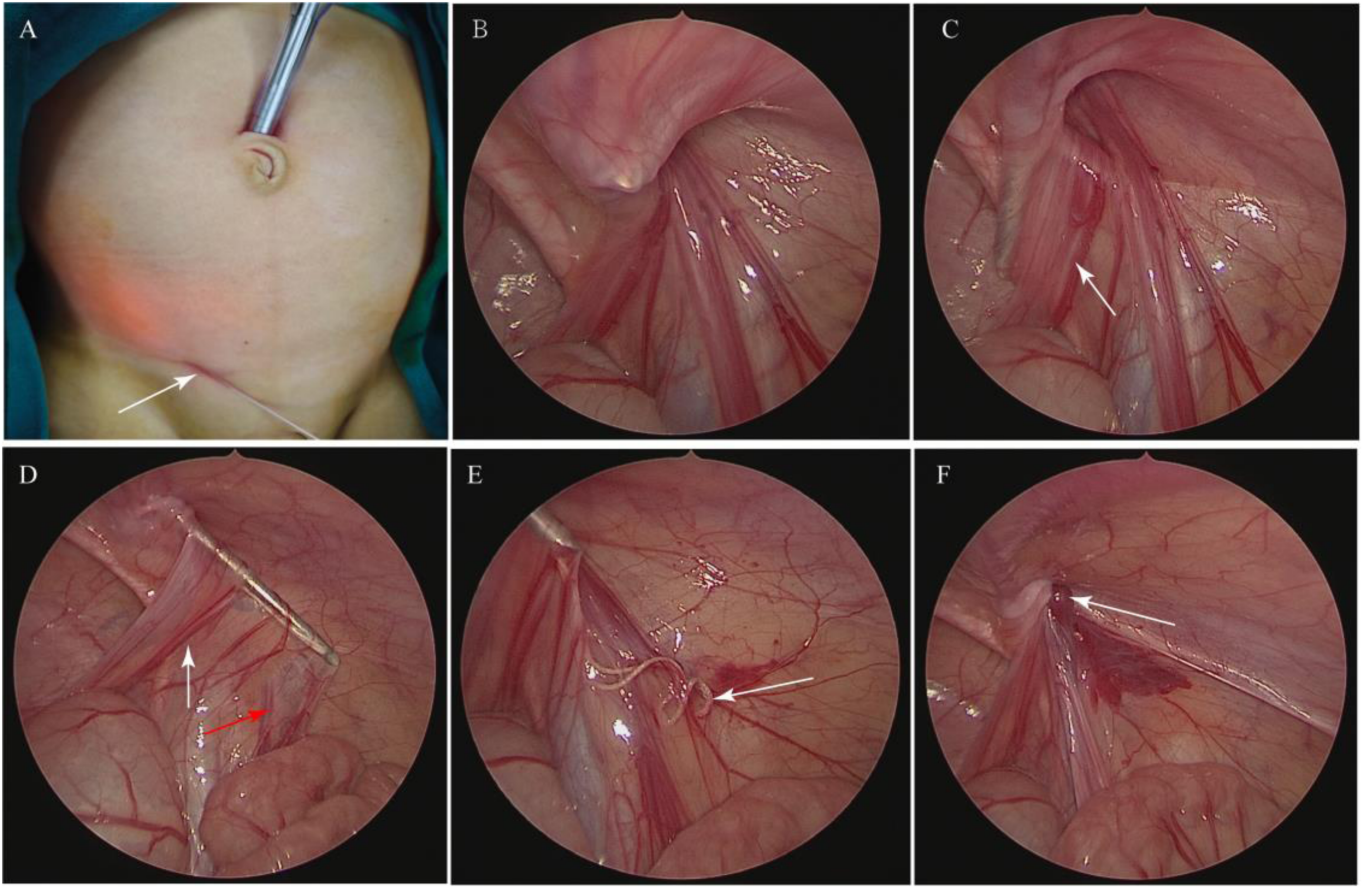
Steps in the LCEC procedure for inguinal hernia. **(A)** A 5 mm incision was made around the umbilicus for the laparoscopic light source, and a 1 mm incision was made at the surface projection point of the inner ring, as indicated by the arrow. **(B)** A hernia needle with 3-0 non-absorbable suture was inserted at the abdominal transverse striation to the extraperitoneal space. **(C)** The hernia needle was passed back to the retroperitoneum and passed through the surface of the vas deferens, indicated by the arrow. **(D)** The hernia needle was passed through the vas deferens, as indicated by the white arrow, and the vessels, as indicated by the red arrow. **(E)** The suture was retained in the extraperitoneal space, as indicated by the arrow. **(F)** Inner inguinal ring closure was performed, as indicated by the arrow.

The LCEC procedure for inguinal hernia was also revised to include one trocar in the umbilicus. A 5 mm incision was made around the umbilicus for the laparoscopic light source, and a 1-mm incision was made at the surface projection point of the inner ring (Figure 2A). Under laparoscopic vision, a hernia needle with a 3/0 non-absorbable suture was inserted at the abdominal transverse striation to the extraperitoneal space, and an identical subcutaneous path was maintained (Figure 2B). The inclined surface of the hernia needle reached back to the retroperitoneum and passed through the surface of the vas deferens (Figure 2C). After the hernia needle was passed through the vas and vessels (Figure 2D), it continued to separate the extraperitoneal space as it was moved forward approximately 2 to 3 cm without puncturing the peritoneum while retaining the suture in the extraperitoneal space which was different with LIC procedure (Figure 2E). The hernia needle was slowly withdrawn along the original path from the extraperitoneal space to the top of the internal inguinal ring and inserted along the lateral half side of the internal ring clamp the suture. Finally, the ligation of the inner inguinal ring was checked by laparoscopy (Figure 2F). After surgery, the umbilical incision sutured by a 5/0 victory suture, and the 1-mm incision on the surface projection point of the inner ring adhered with medical adhesion agent.

### 2.3 Follow-up assessment

All Patient were required two-days hospitalization plan for better evaluate the postoperative pain and ensure the safety of the surgery. Outpatient interviews at 2 weeks, 3 months, 1 year, 3 years, and 5 years after the intervention. Postoperative pain scores were assessed and recorded at 12 and 24 h after surgery using the Children'**s** Hospital of Eastern Ontario Pain Scale (CHEOPS) scoring system, which measures 6 categories of pain behaviors: crying, facial expressions, speech, leg movements, physical activity, and degree of palpability of the wound. Each category is scored from 0 to 2 or from 1 to 3 points, with a total possible score ranging from 4 to 13 points and a total score of less than 6 points indicating no pain. All patients did not use any analgesics for postoperative analgesia. If the patient experiences unbearable pain (usually pain score exceeding 9 points) after surgery, oral ibuprofen can be used for analgesia. All patient complications were recorded, and the presence of wound infection, stitch abscess, and recurrent inguinal hernia was determined, with recurrent inguinal hernia diagnosed on the basis of clinical examination and ultrasound. The position, size, and blood supply of the testes were measured by ultrasound at 2 weeks, 3 months, 1 year, 3 years, and 5 years after surgery.

### 2.4 Outcome measures

The primary outcome measures were success and recurrence rates, which were compared between the LCEC and LIC groups. The secondary outcome measures were surgical duration; length of hospital stay (LOS); postoperative pain score; and rates of postoperative complications, including rate of wound infection, stitch abscess, and testicular atrophy.

Statistical analysis was performed using Statistical Package for the Social Sciences (SPSS) software version 22.0 (SPSS Inc., Chicago, IL, USA). Normally distributed variables were compared using *t* tests and expressed as means ± standard deviations, and non-normally distributed data were analyzed using nonparametric equivalent tests (Mann–Whitney *U* or Kolmogorov-Smirnov tests). Categorical data were compared using the Pearson *χ*^2^ test or Fisher exact test as appropriate and expressed as the percentage frequency. A *P*-value < .05 was considered statistically significant.

## 3 Results

### 3.1 Demographic and clinical characteristics

**Figure 3:**
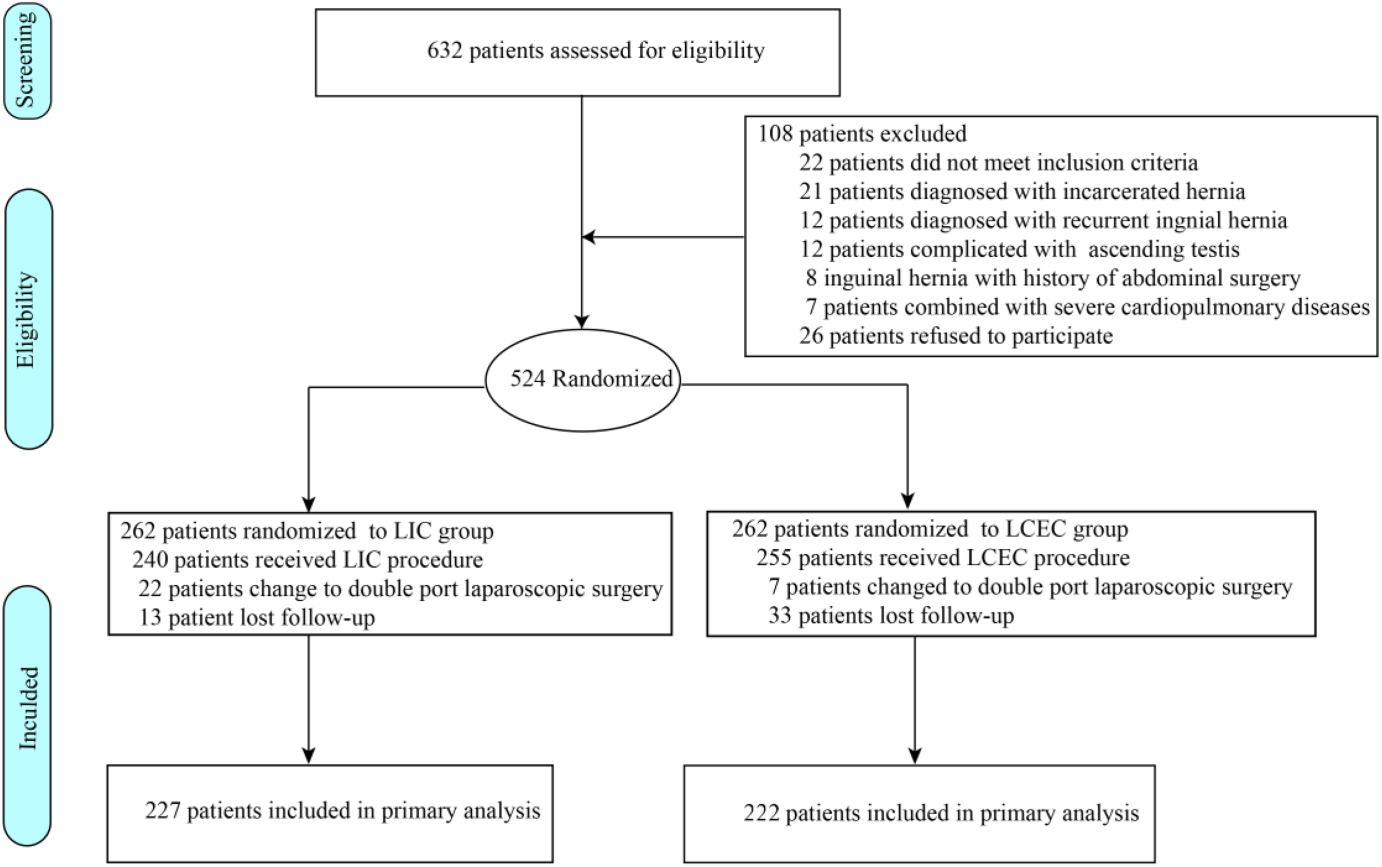
Flowchart of patients' selection.

Figure 3 shows the patient selection and randomization process. From August 2016 to December 2017, 632 patients with inguinal hernia who had been planned to undergo inguinal hernia closure procedures were screened for inclusion. Of the 524 patients who met the inclusion criteria, were 1:1 randomized to LIC group and LCEC group. 22 patients in LIC group and 7 patients in LCEC group underwent double-port laparoscopic surgery because of difficulty in passing the needle through the vas or vessels. Finally, 240 patients (median age, 2.3 ± 1.3 years) were randomized to undergo LIC and 255 patients (median age, 2.5 ± 1.3 years) to undergo LCEC. Of the 46 patients lost to follow-up, 33 were in the LCEC group and 13 in the LIC group, leaving 222 patients in the LCEC group and 227 patients in the LIC group available for primary outcome analysis at 5-year follow-up. The baseline characteristics of the patients who declined to participate were similar to those who underwent randomization with respect to age at surgery, weight at surgery, and type of inguinal hernia.

### 3.2 Primary outcomes

As shown in Table 2, the baseline characteristics of the LIC and LCEC groups were similar (Table 1). Among the 240 patients randomized to the LIC group, the surgical plan of 22 patients was changed to double-port laparoscopic surgery because of difficulty passing the hernia needle through the vas or vessels, resulting in an LIC success rate of 90.8%. Among the 255 patients in the LCEC group, the surgical plan of 7 patients was also changed to double-port laparoscopic surgery because of difficulty passing the hernia needle pass through the vas or vessels, resulting in an LCEC success rate of 97.3%, significantly higher than the LIC success rate (*P* *=* .004). During the 5-year follow-up period, the rate of inguinal hernia recurrence did not significantly differ between the LIC group (2.91%, 7 of 240children) and the LCEC group (0.78%, 2 of 255 children; *P* = .097; Table 2).

**Table 1:** Comparison of patient clinical characteristics.

Characteristic	LIC group (*n* = 240)	LCEC group (*n* = 255)	*P* value
Age at surgery, y (mean ± SD)	2.3 ± 1.3	2.5 ± 1.3	0.088
Weight at surgery, kg (mean ± SD)	12.7 ± 2.9	13.1 ± 2.8	0.119
Type of inguinal hernia			0.851
Right sided, *n* (%)	78 (32.5%)	83 (32.5%)	
Left sided, *n* (%)	93 (38.7%)	104 (40.8%)	
Bilateral, *n* (%)	69 (28.8%)	68 (26.7%)	

**Table 2:** Comparison of primary outcomes.

	LIC group (*n* = 240)	LCEC group (*n* = 255)	*P* value
Operative success, *n* (%)	218 (90.8%)	248 (97.3%)	0.004
Recurrence, *n* (%)	7 (2.91%)	2 (0.78%)	0.097

### 3.3 Secondary outcomes

Table 3 shows the secondary outcomes of the study groups. There was no significant difference in surgical duration between the LIC and LCEC groups (22.68 ± 8.5 vs. 21.8 ± 6.0 min, respectively; *P* = . 205). In contrast, the median CHEOPS score at 12 h postoperatively was significantly lower in the LCEC group than in the LIC group (6.3 ± 1.1 vs. 6.6 ± 1.5, respectively; *P* = .016), as was the median CHEOPS score for groin pain at rest at 24 h postoperatively (5.3 ± 0.9 vs. 5.5 ± 1.1, respectively; *P* = .035). The LOS was significantly shorter in the LCEC group than in the LIC group (1.9 ± 0.6 vs. 2.4 ± 0.7, respectively; *P* < .001), whereas the wound infection rate of 5.7% (13 of 227 children) was significantly higher in the LIC group than the wound infection rate of 0.93% (2 of 222 children) in the LCEC group (*P* = .004). Of the 15 patients who experienced surgical site infections related to delayed healing of the incision, 8 of the 13 patients with wound infections in the LIC group experienced umbilicus incision infection, 5 patients in the LIC group experienced inguinal incision infection, and 2 patients in the LCEC group experienced umbilicus incision infection. The stitch abscess rate of 7.0% in the LIC group was significantly higher than the 1.4% rate in the LCEC group (*P* = .003). Of the 16 patients with stitch abscess at the inguinal site, all needed to undergo redo surgery to remove the inguinal suture. At 5-year follow-up, 3 patients in the LIC group complained of inguinal hernia recurrence. No patients in either group experienced testicular atrophy.

**Table 3:** Comparison of secondary outcomes.

	LIC group (*n* = 227)	LCEC group (*n* = 222)	*P* value
Operative time, min (mean ± SD)	22.68 ± 8.5	21.8 ± 6.0	0.205
CHEOPS score at 12 h (mean ± SD)	6.6 ± 1.5	6.3 ± 1.1	0.016
CHEOPS score at 24 h (mean ± SD)	5.5 ± 1.1	5.3 ± 0.9	0.035
Length of hospital stay, d (mean ± SD)	2.4 ± 0.7	1.9 ± 0.6	<0.001
Wound infection, *n* (%)	13 (5.7%)	2 (0.93%)	0.004
Stitch abscess, *n* (%)	16 (7.0%)	3 (1.4%)	0.003
Testicular atrophy, *n* (%)	0	0	

## 4 Discussion

Laparoscopic inguinal hernia repair in children, whether by a transabdominal or a complete extraperitoneal procedure, has been available as an alternative to open repair since the early 1990s (10). Having the advantages of less scarring and more rapid recovery, laparoscopic procedures include laparoscopic intra-abdominal suture of the inner ring and laparoscopic percutaneous ligation of the extraperitoneal inner ring. Laparoscopic intra-abdominal inner ring suture mainly includes three-port, double-port, and single-port methods (11), and laparoscopic percutaneous ligation of the extraperitoneal inner ring includes single-port and double-port laparoscopy (12). Compared with laparoscopic intra-abdominal inner ring suture, laparoscopic percutaneous ligation of the extraperitoneal inner ring is relatively simple and requires a shorter learning curve. Recent examination of the practice of single-port laparoscopy has shown that it can significantly reduce operative time, yield satisfactory efficacy, and decrease recurrence rate, leading it to become common in clinical practice compared with traditional laparoscopic inguinal hernia closure (11). Several studies have reported that the hernia needle combined with single-port laparoscopy can be used to repair inguinal hernia in children with good results (13). However, to date, no study has compared the effectiveness of intracorporeal and completely extracorporeal procedures under single-port laparoscopy.

The LIC procedure for inguinal hernia in children includes two steps: separation of the peritoneum and vas deferens or spermatic vessels, which eases the passage of the needle through the vas deferens or spermatic vessels and sewing the suture into the abdominal cavity after puncturing the peritoneum. In our study, 22 patients had to undergo double-port laparoscopic surgery because of difficulty in passing the needle through the vas or vessels. However, LIC can increase damage to the peritoneum because of the need to puncture the peritoneum and sew the suture into the abdominal cavity after pushing it through the vas deferens and then gliding it through the spermatic vessels. After the needle is passed through the abdominal cavity along the holes made in the peritoneum, the silk thread is hooked and brought outside of the body before the inner ring is closed. Although LIC can effectively resolve the difficulty of passing the needle through the vas deferens and spermatic cord, the entire operation cannot be performed at the extraperitoneal site, which can affect the success and complication rates of LIC.

In our study, we introduced an LCEC procedure that permits complete extraperitoneal closure under single-port laparoscopy. Although we found that the success rate of our LCEC procedure was significantly higher than that of LIC, the success of LCEC depends on performing several steps. First, the bevel at the tip of the hernia needle should be as close to and parallel to the peritoneum as possible to avoid rotation of the needle and increase the rate of puncturing the peritoneum. Second, if there are many folds in the medial peritoneum or the vas deferens is in the deep surface of the iliac blood vessels, the hernia needle should be passed for a long distance in the direction of the medial lower bladder, and then the peritoneum should be punctured and the vas deferens exposed to the greatest extent possible. The hernia needle should be held against the vas deferens in the direction of the outer lower side of the inner ring orifice. If the vas deferens is close to the peritoneum and the hernia needle cannot be entered through it at one pass, a tunnel can be created behind the vas deferens to expand the extraperitoneal space before returning the fairy needle to the vas deferens and attempting to pass the needle through the vas deferens again. Third, the hernia needle should be aimed toward the proximal end of the iliac vein and the distal end of the spermatic vein while remaining close to the peritoneum and closely following the surface of the spermatic vessels. It should then be passed around the iliac fossa outside the peritoneum so that it can easily be passed through the spermatic vein. Fourth, when the hernia needle is retracted to the top of the inner ring orifice, the bevel of the hernia needle should be prevented from coming out of the peritoneum. It is important to remain aware that reinserting the hernia needle may increase the number of puncture points, thus causing postoperative hydrocele. Fifth, ligating extraperitoneal fat and abdominal wall muscles into the suture knot should be avoided to prevent difficulty in pulling out the needle, as the temple needle is filled with adipose tissue. If this occurs, the fox needle should be repeatedly advanced and retreated in the direction of the iliac fossa. In addition, the testicles should be pulled to the scrotum and the inguinal gas exhausted before the closure of the inner ring.

In previous studies, postoperative analgesia duration was reported to be over 300 min (14, 15). Therefore, in our study, the CHEOPS scores were assessed at 12 and 24 h postoperatively, which were 6.3 and 5.3, respectively, for the LCEC group, significantly lower than those of the LIC group. Compared with LIC, LCEC decreases postoperative pain because it does not puncture or damage the peritoneum, thereby preserving the absolute integrity of the peritoneum. Other advantages include fewer separation surfaces, smaller incisions, less postoperative pain, and more rapid recovery, making it suitable for performing at a day surgery center.

Regarding perioperative complications of LCEC and LIC, intraoperative bleeding, postoperative inguinal swelling, ascending testes, testicular atrophy, and surgical site infection have been reported (16). In this study, wound infection and stitch abscess were the main complications and the main cause of recurrence during follow-up, with no postoperative testicular atrophy observed for either procedure. The criteria we included for umbilical infection were postoperative redness, swelling, and exudation of the umbilical incision. Our incidence of umbilical infection is higher than other literatures reported (1, 6, 7), which may be related to our inclusion criteria. We have found that the umbilical infection can be improved through traditional conservative dressing changes. In the early stage of our study, wound infection was mainly caused by umbilical incision infection. The incidence of umbilical incision infection in LIC procedure is greater than LCEPC procedure, which may be related to 22 patients have to switch operative method from single-port to double-port laparoscopic procedure in LIC group. At the same time, many patients suffered improper incision care after going back home when achieved daytime surgery management. Stitch abscess, which often occurs at 6 months to 2 years after inguinal hernia closure, is present in the inguinal area where the hernia sac is ligated. Stitch abscess may be related to several factors, including the use of non-absorbable sutures, which may increase the risk of suture reactions, and ligation of the abdominal wall muscles or adipose tissue into the knot, leading to inflammation and groin pain. Requiring removal of the ligated suture, stitch abscess is a main reason for recurrence of postoperative inguinal hernia. In our study, 4 children in the LIC group and 2 children in the LCEC group experienced postoperative recurrence of inguinal hernia due to stitch abscess.

This study has several limitations. Firstly, as a prospective single-center RCT, this study would have ideally compared two completely different operations and have controlled for the variable of age, neither of which it did. Secondly, the findings may reflect surgeon experience bias, as the experience of different surgeons with LIC to LCEC over time may have generated differences in patient outcomes.

In conclusion, the results of this RCT comparing LIC to LCEC for pediatric inguinal hernia indicate that LCEC is a safe and effective procedure for repairing inguinal hernia, showing the potential to increase the surgical success rate while decreasing groin pain, and incidence of postoperative complications.

## Data Availability

The original contributions presented in the study are included in the article/Supplementary Material, further inquiries can be directed to the corresponding author.
